# *In vitro* antibacterial activity and *in vivo* efficacy of hydrated clays on *Mycobacterium ulcerans* growth

**DOI:** 10.1186/s12906-016-1020-5

**Published:** 2016-01-30

**Authors:** Sarojini Adusumilli, Shelley E. Haydel

**Affiliations:** 1 Biodesign Institute Center for Infectious Diseases and Vaccinology, Arizona State University, Tempe, Arizona USA; 2 School of Life Sciences, Arizona State University, Tempe, Arizona USA

**Keywords:** *Mycobacterium ulcerans*, Buruli ulcer, Therapeutic, Clays, Antibacterial, *In vivo*

## Abstract

**Background:**

Buruli ulcer, caused by *Mycobacterium ulcerans*, is a localized skin lesion that can progress to extensive ulceration and necrosis if left untreated. Unpublished studies of hydrated clays for therapeutic, topical treatment of Buruli ulcer suggest that specific clay mineral products may have beneficial effects on wound healing. In this study, we evaluated the *in vitro* antibacterial activity of a panel of clay mixtures and their derivative leachates against *M. ulcerans* and assessed the *in vivo* efficacy of topically-applied, hydrated clays on Buruli ulcer progression in mice infected with *M. ulcerans.*

**Methods:**

*M. ulcerans* 1615 was incubated with 10 % suspensions of CB07, CB08, CB09, CB10, and BY07 clay mixtures, and survival was determined over 28 days. For animal experiments, we examined the effect of topical hydrated clay therapy on Buruli ulcer progression *in vivo* in mouse tails subcutaneously infected with *M. ulcerans* 1615*.*

**Results:**

The CB07, CB08, and CB09 clays exhibited bactericidal activity against *M. ulcerans* after 7, 14, 21, and 28 days of incubation. In contrast, clay leachates exhibited inhibitory, bacteriostatic effects on *M. ulcerans* growth *in vitro*. After establishing an ulcerative *M. ulcerans* infection for three months, ulcerated regions of the tails were treated once daily (five consecutive days per week) for 22 days with hydrated CB09 clay poultices. Mice in the clay treatment group exhibited healing as assessed by gross morphological changes and a reduction in *M. ulcerans* present in the wounds.

**Conclusions:**

These data reveal that specific clays exhibit *in vitro* bactericidal activity against *M. ulcerans* and that hydrated clay poultices may offer a complementary and integrative strategy for topically treating Buruli ulcer disease.

## Background

Buruli ulcer, a skin disease caused by *Mycobacterium ulcerans*, is the world’s third most common mycobacterial infection after tuberculosis and leprosy [1]. The macrolide exotoxin, mycolactone, is a key *M. ulcerans* virulence factor and plays a central role in Buruli ulcer pathology [2]. After *M. ulcerans* infection is established, Buruli ulcer appears as a painless nodule in the skin. Left untreated, *M. ulcerans* destroys skin, subcutaneous fat, and sometimes bone [3–6]. Gradually, an ulcer develops with a necrotic center and undermined edges, surrounded by an indurated area [6–8].

Buruli ulcer occurs primarily in resource-poor areas where limited access and the cost of seeking health care can be prohibitive. Given that the disease symptoms are generally painless, some patients do not seek treatment until the disease has progressed to the ulcerative stage [1, 3, 9]. *M. ulcerans* infection can be effectively managed with a combination antibiotic therapy of rifampicin and streptomycin or rifampicin and clarithromycin [10–12]. Lesions may also spontaneously heal by unknown mechanisms [1, 8]. In the ulcerative stage of disease, *M. ulcerans* infection is generally treated by surgical removal of infected and surrounding healthy skin and tissue, pre- and post-surgical antibiotic administration, and subsequently may require grafting of healthy skin tissue [13]. Cure from the disease is expensive, time consuming, causes scarring and tissue damage, and may result in deformities and disabilities. Moreover, despite free of charge biomedical care and support for Buruli ulcer patients in Cameroon, non-medical costs, productivity losses, and social isolation cause patients to abandon in-patient hospitalization and medical treatment [14]. A topical, therapeutic strategy that complements existing treatment options and is socially acceptable in parts of the world where Buruli ulcer is problematic would be desirable [14, 15].

Previously, topical application of hydrated clay poultices was observed to be effective in treating and promoting healing of infected tissue in Buruli ulcer patients [16]. In the uncontrolled, observational study, either green illite or green montmorillonite clays were hydrated and applied daily as a 2-cm thick poultice with an overlay of 10-30 cm beyond the ulcer. After months of topical treatment, the ulcers were minimized, and the infections often healed with some soft tissue scarring [16]. Haydel et al. [17] subjected the two clay minerals used in the above study to *in vitro* antimicrobial susceptibility testing, demonstrating effects ranging from enhanced microbial growth to complete growth inhibition of several pathogenic and non-pathogenic bacteria. These patient observations [16] and *in vitro* microbiological data [17] imply that topical application of hydrated clays or other absorptive materials could potentially be an effective, inexpensive, and complementary treatment option for Buruli ulcer. However, the effect of hydrated clays on *M. ulcerans* growth *in vitro* or during *in vivo* infection remains unknown. In this study, we examined the effect of different clay mixtures and their derivative leachates on *M. ulcerans* growth *in vitro*. We also examined the effect of antimycobacterial clay treatment on Buruli ulcer progression *in vivo* in a mouse tail model of infection.

## Methods

### *M. ulcerans* growth

*M. ulcerans* 1615 was obtained from Dr. Pamela Small (University of Tennessee, Knoxville, TN, USA). Bacteria were grown on Middlebrook 7H10 agar (Difco) supplemented with 10 % oleic acid-bovine serum albumin-dextrose-catalase (OADC), 0.05 % Tween 20, and 0.5 % glycerol (supplemented M7H10 agar) at 32 °C. Bacterial inocula were serially passaged through a 25-guage needle to disrupt mycobacterial clumps, diluted in PBS (pH 7.4) to obtain the desired concentration based on OD_600_ measurements, and quantified by plating on supplemented M7H10 agar to determine viable colony forming units (CFU).

### Clay mixtures and clay mixture leachate preparation

Antibacterial, mineralogical, and chemical characteristics of the CB07, CB08, CB09, CB10, and BY07 clay mixtures and derived leachates have been previously described [18–20]. The CB clay mixtures are all composed of approximately 52 % clays and 48 % non-clay minerals [20]. The most abundant clay mineral in all of the CB samples is illite-smectite (36–37 %), followed by montmorillonite (9.7–14.2 %) and kaolinite (1.4–3.6 %). The most abundant non-clay mineral present in the CB samples is quartz (34–37.3 %), followed by pyrite (4–5.5 %) and jarosite (2.4–4.7 %) [20]. The most abundant minerals present in the BY07 clay mixture are Ca-smectite clay (37.3 %), followed by anorthoclase feldspar (23.0 %) and quartz (13.7 %) [19]. All clay mixtures were sterilized by autoclaving for 1 h at 121 °C prior to experimental use. Derived leachates were obtained by vigorously stirring clay mixtures (1 g/20 ml) in sterile, deionized H_2_O (dH_2_O) for 24 h. Subsequently, the hydrated clay mixture suspensions were centrifuged (31,000 × g) for 3 h at 4 °C to separate insoluble and soluble fractions. The aqueous supernatant (leachate) was collected and sterilized by passage through a 0.22 μm filter.

### *In vitro* antimicrobial susceptibility testing

*M. ulcerans* 1615 were exposed to CB07, CB08, CB09, CB10, and BY07 clay mixtures or derived leachates [18, 19], and survival was determined by enumerating colonies on supplemented M7H10 agar plates. Briefly, 100 mg of sterilized clay minerals were added to late-logarithmic phase *M. ulcerans* in supplemented M7H9 broth (1 ml), and the bacteria-clay suspensions were incubated at 32 °C on a rotating drum. To assess antimicrobial activity of leachates, late-logarithmic phase *M. ulcerans* 1615 was pelleted, resuspended in 1 ml of CB07, CB08, CB09, CB10, or BY07 clay leachates, water, or supplemented M7H9 broth, and incubated at 32 °C on a rotating drum. Experimental samples and controls were collected on 0, 7, 14, 21, and 28 days, subjected to 10-fold serial dilutions, plated on supplemented M7H10 agar, and incubated at 32 °C to determine viable CFU. Bactericidal activity is defined as the ability to kill bacteria, while bacteriostatic activity means that the agent prevents the growth of bacteria. Minimal bactericidal concentration (MBC) is defined as the lowest concentration of a particular antibacterial agent that kills ≥ 99.9 % of the bacterial population in a liquid medium.

### Bacterial inoculum preparation for *in vivo* studies

To determine the *M. ulcerans* 1615 inoculum required to establish a visible ulcer, groups of four female BALB/c mice, aged four to six weeks, were injected in the tail with 10^3^, 10^5^, or 10^7^ CFU of *M. ulcerans* in 30 μl PBS. Aliquots of *M. ulcerans* 1615 inoculum, grown in supplemented M7H9 broth to late-logarithmic phase of growth, were prepared as described above.

### Animal infection procedures and bacterial enumeration of *M. ulcerans*-infected mouse tails

A total of 30 four- to-six week old female BALB/c mice (Charles River Laboratories) were subcutaneously injected in the tail with ~10^6^ *M. ulcerans* 1615 cells in 30 μl of PBS. During *M. ulcerans* inoculation, the mice were anesthetized by intraperitoneal injection of 0.05 ml per 25 g of body weight with a mixture containing 21 mg ketamine, 2.4 mg xylazine, and 0.3 mg acepromazine. Five additional mice injected with PBS served as negative controls. On day one post inoculation, five mice infected with *M. ulcerans* were euthanized, and the tails were used to enumerate *M. ulcerans* in the infected animals. Briefly, tails were minced with blades, ground with a Potter-Elvehjem homogenizer in 0.15 M NaCl, and decontaminated with an equal volume of N-acetyl-l-cysteine sodium hydroxide. Aliquots of serial dilutions were plated on supplemented M7H10 agar, and the plates were incubated at 32 °C for 8-10 weeks. Mice were monitored daily. Of the 25 remaining animals infected with *M. ulcerans*, only 15 animals developed a visible ulcer. Five animals were euthanized to enumerate bacterial load before treatment, and the remaining 10 animals were either treated with hydrated clay poultices (n = 5) or left untreated (n = 5). All animal experiments were carried out in strict accordance with the recommendations in the Guide for the Care and Use of Laboratory Animals of the National Institutes of Health. All animal procedures were approved by the Arizona State University Animal Care and Use Committee (protocols 09-1030R and 12-1240R) and conducted according to relevant national and international guidelines.

### Topical application of hydrated clay poultices

Approximately three months postinfection, mice were either treated with hydrated clay at the site of infection or cleansed with sterile saline (no treatment). Mice in the clay treatment group (n = 5) were treated with hydrated CB09 clay (~0.2 grams clay mixed with 100 μl sterile dH_2_O) at the site of *M. ulcerans* infection. During topical application of hydrated clay poultices, mice were restrained using a commercial acrylic mouse restrainer. The infected area of the tail was initially washed with cotton swabs dipped in sterile dH_2_O. Subsequently, a sterile cotton swab was used to evenly apply a thick poultice of hydrated clay to the site of infection. Infected and treated tails were then covered with Tegaderm waterproof transparent dressings followed by an adhesive bandage to prevent the mice from disturbing and removing the clay poultice. For five consecutive days per week, the Tegaderm dressing and adhesive bandages were removed, and the infected tails were cleansed with sterile dH_2_O. After gentle washing to remove the clay poultice, freshly hydrated clay was applied, and the tails were redressed with bandages. Mice in the no treatment group (n = 5) did not undergo any treatment at the site of *M. ulcerans* infection. Similar to the infected and treated animals, the infected area of the tails of mice in the no treatment group was subjected to gentle washing with sterile water and subsequent redressing with Tegaderm and an adhesive bandage for five consecutive days per week. Decreased erythema and ulceration and reduction in bacterial load were considered indicators of wound healing. Two animals, one in the clay treatment group and one in the no treatment group, developed secondary infections and were euthanized prior to the end of the study. After 22 days of treatment, the remaining eight mice were euthanized. To enumerate bacterial load, the tails of the euthanized animals were removed aseptically, weighed, and homogenized as described above. The number of viable *M. ulcerans* cells in the tissue was determined by plating serial dilutions on supplemented M7H10 agar. The CFU were counted after 8-10 weeks of incubation at 32 °C and expressed as CFU/g tissue.

### Statistical analyses

We used ANOVA with Tukey’s or Dunnett’s multiple comparisons to assess statistical significance. Each *P* value was adjusted to account for multiple comparisons of mean values across groups. Data were analyzed using GraphPad Prism 6.

## Results

### Clays are bactericidal to *M. ulcerans in vitro*

**Fig. 1:**
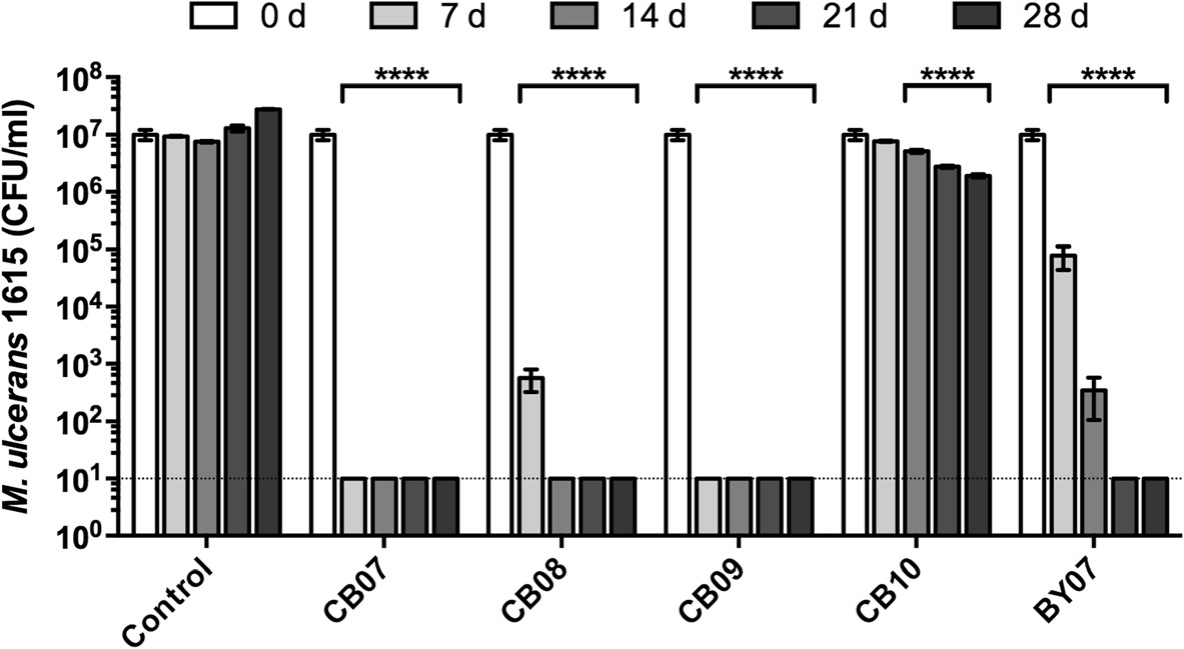
Effect of clay mixtures on *M. ulcerans* growth *in vitro. M. ulcerans* was incubated with a 10 % suspension of clay in supplemented M7H9 broth at 32 °C. After 7, 14, 21, and 28 d, survival was determined by plating samples on supplemented M7H10 agar and incubation at 32 °C to determine viable CFU. *M. ulcerans* growth in supplemented M7H9 broth is shown as a control. Data represent three independent experiments performed in triplicate, and results are expressed as mean ± SEM. ****, *p* < 0.0001; ANOVA, Tukey’s multiple comparisons

A 10 % suspension of clays was tested for *in vitro* bactericidal activity against *M. ulcerans*. Of the five different clays tested, CB07 and CB09 exhibited complete *M. ulcerans* bactericidal activity within 7 days of exposure, while CB08 required 14 days of exposure to yield complete bactericidal activity (Fig. 1). BY07 required 21 days to demonstrate complete bactericidal activity, while exposure to CB10 only minimally inhibited *M. ulcerans* growth over 28 days (Fig. 1).

### Effect of aqueous clay leachates on *M. ulcerans* growth *in vitro*

**Fig. 2:**
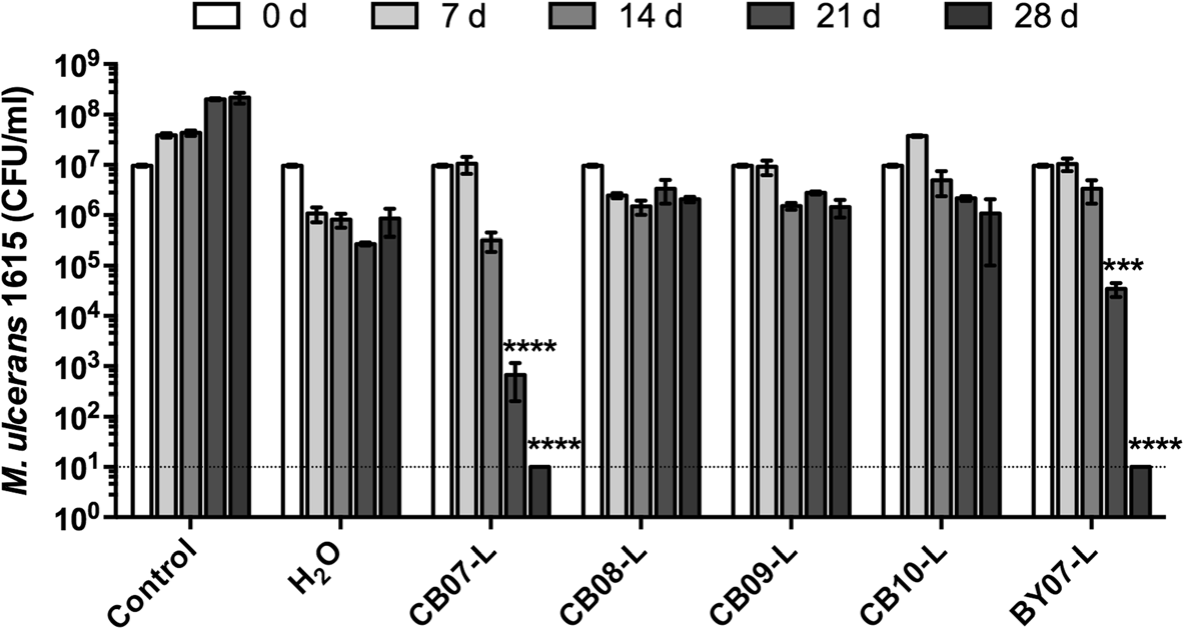
Effect of aqueous clay leachates on *M. ulcerans* growth *in vitro. M. ulcerans* was exposed to 1 ml of supplemented M7H9 broth (control), sterile deionized H_2_O, or clay leachates. At specified times, samples were plated on supplemented M7H10 agar and incubated at 32 °C to determine viable CFU. Data represent three independent experiments performed in triplicate, and results are expressed as mean ± SD. Statistically significant differences, compared to dH_2_O-incubated samples on corresponding days, are shown. ***, *p* < 0.001; ****, *p* < 0.0001; ANOVA, Tukey’s multiple comparisons

To differentiate the bactericidal effect of the soluble ions released from the clay particles, we examined the effect of aqueous leachates on growth of *M. ulcerans*. Leachates, which contain water-leachable components of the clay mixtures, were prepared and assayed for antibacterial activity against *M. ulcerans*. Compared to *M. ulcerans* growth inhibition during water incubation, CB08 leachate (CB08-L), CB09 leachate (CB09-L), and CB10 leachate (CB10-L) did not exhibit inhibitory activity over 28 days, and in some cases, incubation with the leachates enhanced *M. ulcerans* viability (Fig. 2). In contrast, incubation with CB07 leachate (CB07-L) and BY07 leachate (BY07-L) resulted in increasing and significant bactericidal activity (*p* < 0.0001) over 28 days (Fig. 2).

### Development and progression of *M. ulcerans* in mouse tails

A pilot study was conducted to determine the optimal inoculum of *M. ulcerans* that would induce a visible ulcer in mouse tails. BALB/c mice were infected with 7.9 × 10^3^, 1.0 × 10^5^ CFU, or 1.6 × 10^7^ *M. ulcerans* CFU. By the second week of infection, all three groups of animals developed swelling and erythema in the tails around the area of inoculation. Although the swelling subsided after the third week of infection, there was persistent erythema in all three groups. By 36 days postinfection, mice in the 10^5^ and 10^7^ groups developed small pinpoint ulcers on their tails. By three months postinfection, mice in the 10^7^ inoculum group developed the most pronounced ulcers.

### Therapeutic effect of hydrated clay on Buruli ulcer progression in mice

**Fig. 3:**
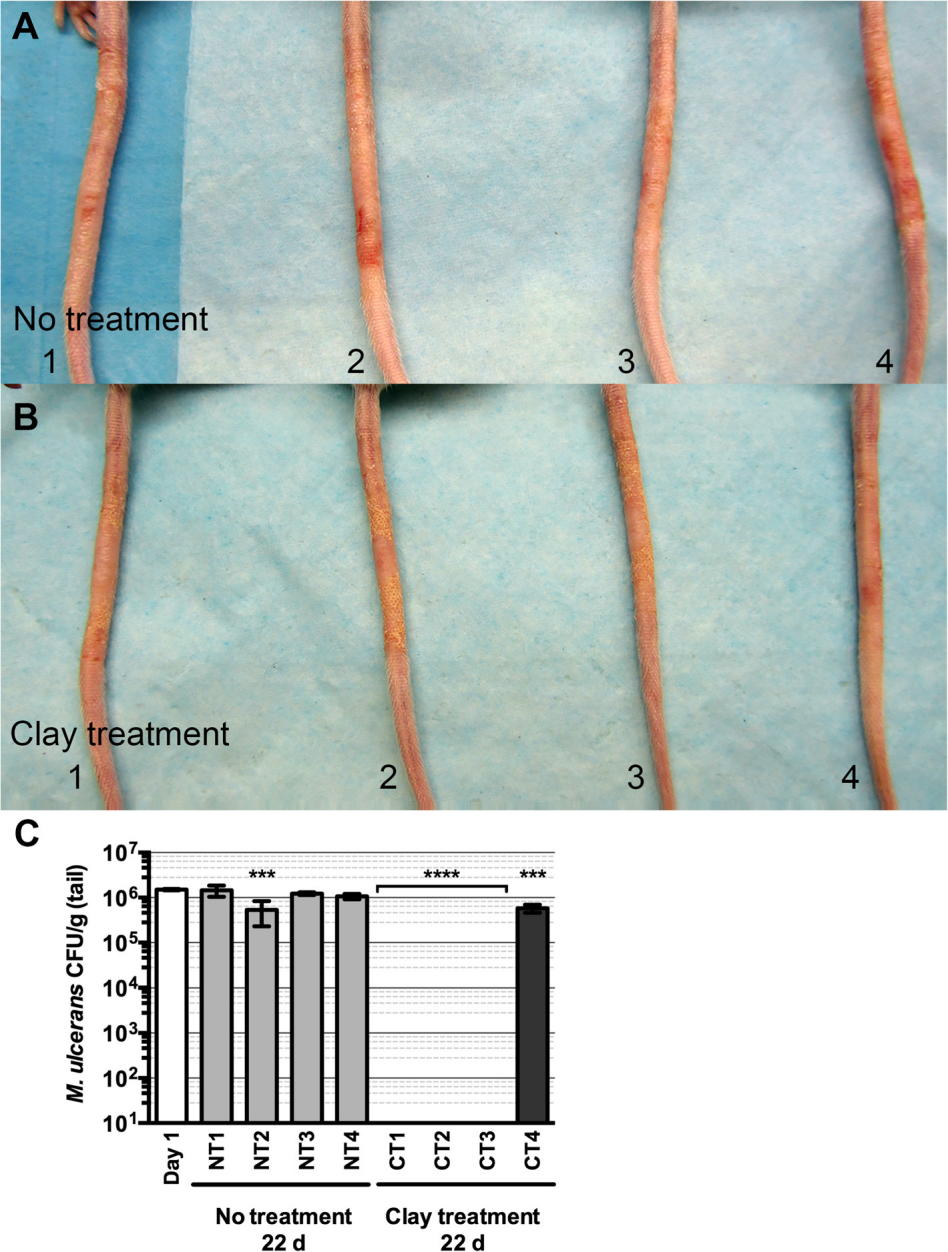
Gross morphology and bacterial detection of *M. ulcerans*-infected mouse tails with and without topical application of hydrated clay mixtures. Disease advancement and gross morphology of *M. ulcerans*-infected tails following 22 d of daily cleansing with sterile dH_2_O (no treatment) (**a**) or daily topical applications of hydrated antibacterial CB09 clay (**b**). After 22 d of topical treatment with the hydrated CB09 clay mixture, *M. ulcerans* survival was determined by collecting the tails from infected mice, processing the samples as described in the methods, and inoculating supplemented M7H10 agar to determine viable CFU per gram of tissue for each animal sample (**c**). Error bars represent the SD from three viable CFU determinations for each animal sample. The average (± SD) number of *M. ulcerans* cells, as determined from five infected animals on day 1 post-infection, is shown for comparison. The limit of detection is 10 CFU. NT, no treatment; CT, clay treatment. ***, *p* < 0.001; ****, *p* < 0.0001; ANOVA, Tukey’s multiple comparisons

Since the clays had a bactericidal effect on *M. ulcerans* growth *in vitro*, we examined the effect of hydrated clay on progression of *M. ulcerans* infection *in vivo*. BALB/c mice were subcutaneously infected in the tails with an inoculum of 1.5 × 10^6^ *M. ulcerans* CFU. By the second week of infection, mice developed swelling and erythema in the tails around the area of *M. ulcerans* inoculation. Although the swelling subsided after the third week of infection, there was persistent erythema in tails of all infected mice. During the course of treatment, one mouse from the clay treatment group was euthanized during the second week due to a secondary infection resulting in purulent erythema of the tail. Similarly, one mouse from the no treatment group was euthanized in the fourth week due to a secondary infection. Approximately three months post infection, affected areas of the mouse tails were either left untreated (no treatment group, n = 4) or treated with hydrated CB09 clay (clay treatment group, n = 4) for 22 days (Fig. 3). As seen in Fig. 3, mice in the no treatment group (panel a) had increased erythema and progression of ulcer when compared to the clay treatment mice (panel b). In the clay treatment group, three of the four mice had decreased erythema (Fig. 3). Tails collected from three of the four mice in the clay treatment group yielded no *M. ulcerans* growth after plating on supplemented M7H10 medium and incubation at 32 °C for eight weeks (Fig. 3c). In contrast, *M. ulcerans* was detected in the tails of untreated mice (Fig. 3c).

## Discussion

The World Health Organization recommends combination antibiotic therapy of rifampicin and streptomycin or rifampicin and clarithromycin with or without surgery (mainly debridement and skin grafting) for treating Buruli ulcer [12]. Since there is currently no specific vaccine against Buruli ulcer [21], an effective topical treatment option that is cost-friendly and easy to use could be welcomed in resource-poor settings where Buruli ulcer is endemic [14, 22]. Several topical therapeutic options for Buruli ulcer have been investigated, including 40 °C heat therapy, phenytoin, clay bandages, and creams containing nitrogen oxides [16, 23–27]. Heat treatment and creams delivering topical nitrogen oxides promoted healing in Buruli ulcer patients [23, 24, 28]. Nitric oxide was also shown to kill *M. ulcerans in vitro* [24, 25]. When applied therapeutically, phenytoin powder did not kill *M. ulcerans* but promoted healing through acceleration of fibrogenesis [27]. Hyperbaric oxygen at a partial pressure of 2.5 kPa was beneficial in healing mice infected with *M. ulcerans* [29].

In an uncontrolled and unpublished study, Buruli ulcer patients at a treatment center in Zouan-Hounien, Ivory Coast were treated with hydrated clay minerals [16]. The described debridement phase focused on hydrated illite clay-mediated removal of necrotic tissue and cleansing of the surrounding tissues. In the scarring phase, a hydrated poultice of montmorillonite clay was applied to the wounds and appeared to promote tissue regeneration [16]. After several months of clay applications, Buruli ulcer wounds were reportedly healed in most of the patients [16]. However, clay therapy was not further investigated in a controlled clinical trial.

In the present study, we investigated the effect of antibacterial clay minerals and clay aqueous leachates on *M. ulcerans* growth *in vitro* and the therapeutic effect on Buruli ulcer progression *in vivo* in a mouse model of *M. ulcerans* infection. Two of the five clays tested exhibited complete *in vitro* bactericidal activity against *M. ulcerans* within 7 days, while two clay mineral leachates demonstrated complete *in vitro* bactericidal activity within 28 days. In contrast, compared to incubation in water, some of the leachates appeared to provide a nutritional benefit to *M. ulcerans*. While the four CB samples are mineralogically similar, there are notable differences associated with the composition of ions bound to their surfaces [20]. For example, exchangeable copper concentrations ranged from 0.04 mM (CB09-L), 0.10 mM (CB08-L), 0.19 mM (CB10-L) to 3.18 mM (CB07-L), an approximate 80-fold difference in concentration across the four samples [20]. While we have established that clay-mediated killing of *Escherichia coli* and methicillin-resistant *Staphylococcus aureus* (MRSA) is attributed to toxicity associated directly with released metal ions (Fe^2+^, Cu^2+^, and Zn^2+^) in an acidic environment [20, 30], it is unknown if these ions are important for killing *M. ulcerans in vitro*. Moreover, while 10 % suspensions of the CB clays completely killed *E. coli* and MRSA *in vitro* within 24 h [20], the CB10 clay (10 % suspension) reduced viability by ~1 log_10_ after 28 d, but did not completely kill *M. ulcerans* (Fig. 1). Further investigations associated with the chemical differences of the CB clays could provide some insight into why the CB10 clay exhibits minimal antibacterial activity against *M. ulcerans*.

The effects of hydrated clay minerals on *M. ulcerans* growth and wound progression in infected mice are likely due to a combination of both physical and chemical characteristics of clay minerals. Clays are small phyllosilicate minerals that are commonly grouped into three main classes – smectite, illite and kaolinite – based on their overall crystal layer structure and expandability. Smectite minerals, which include montmorillonite clay, exhibit high expansion (swelling) capabilities in the presence of water [31]. Conversely, illite and kaolinite are non-expanding clays [31]. We have previously assessed the ability of different hydrated clays to treat MRSA-infected superficial, cutaneous wounds in mice [32]. We demonstrated that natural and ion-exchanged illite clays performed best, as measured by bacterial load, inflammatory response and gross wound morphology with significant decreases in bacterial viability and dermatitis [32]. Topical application of kaolinite clay was the least effective, resulting in the lowest decrease in bacterial load and exhibiting severe dermatitis [32]. Although the CB09 clay mixture used in this study is a heterogeneous mixture of illite-smectite, montmorillonite, kaolinite, and non-clay minerals with different structural and expansion properties [32], daily application and removal of the clay poultices could diminish *M. ulcerans* growth due to an unfavorable chemical environment and could result in physical removal of *M. ulcerans* and/or mycolactone via adsorption. Mycolactone plays a central role in the pathogenesis of *M. ulcerans* and is thought to be responsible for most of the tissue destruction that occurs in a Buruli ulcer wound [2]. In macrophages, fibroblasts, and epithelial and endothelial cells, mycolactone blocks Sec61-dependent protein translocation into the endoplasmic reticulum, leading to degradation of aberrantly-located proteins [33], so reduction or removal of mycolactone during daily application and removal of clay poultices would be beneficial for promoting a proinflammatory response and wound healing.

Notably, there are several limitations of this study. The number of animals developing ulcers and available for topical treatment was small. Of the 25 animals infected with *M. ulcerans*, only 15 animals developed a visible ulcer within two to three months postinfection, suggesting that the 10 mice without a visible ulcer or lesion were insufficiently inoculated. After euthanizing five mice for quantitative assessment of *M. ulcerans* present in the tail wounds, 10 mice were available for treatment. Subsequently, one mouse in the no treatment group and one mouse in the clay treatment group developed superinfections, so only four mice remained in each group. While the three mice in the clay treatment group were negative for *M. ulcerans* growth, homogenate samples were not tested by PCR. Previous studies indicate that human tissue specimens deemed culture negative yielded positive PCR results, suggesting persistance of mycobacterial material [10, 34]. In the absence of PCR testing, it is unknown if mycobacterial material persisted in the mouse tail samples deemed culture negative. As determined by clinical improvement and reduction of bacterial burden, three mice were successfully treated with CB09 clay poultices. However, in the absence of systemic antibiotic therapy, it is possible that disease recurrence could occur at the original site of infection. A longer study is necessary to investigate recurrence-free healing of Buruli ulcer after topical treatment with hydrated antibacterial clay.

## Conclusions

Several clays with demonstrated antibacterial activity against Gram-negative and Gram-positive pathogens also exhibit *in vitro* bactericidal activity against *M. ulcerans*. Mice treated with hydrated antibacterial clay healed *M. ulcerans* lesions *in vivo*, as assessed by changes in gross morphology and bacterial enumeration, suggesting that clay minerals or other similar adsorptive/absorptive materials may be of value in the complementary and integrative management of Buruli ulcer lesions. Since natural, mineralogically-identical clay samples exhibit variability in chemical composition and *in vitro* antibacterial activity [20], we are pursuing customized and nanostructured porous, absorptive materials, which offer standard composition and controlled antibacterial efficacy, for topical treatment of *M. ulcerans* infections.
